# Supplementary material for: The causal relationship between the human gut microbiota and pyogenic arthritis: a Mendelian randomization study
Source: Front Cell Infect Microbiol. 2024 Nov 26;14:1452480. doi: 10.3389/fcimb.2024.1452480 (PMC11629706; doi:10.3389/fcimb.2024.1452480)
Supplement: Supplementary file 1 [file DataSheet1.zip › mendelian test/ebi-a-GCST90027567.csv_forest.pdf]

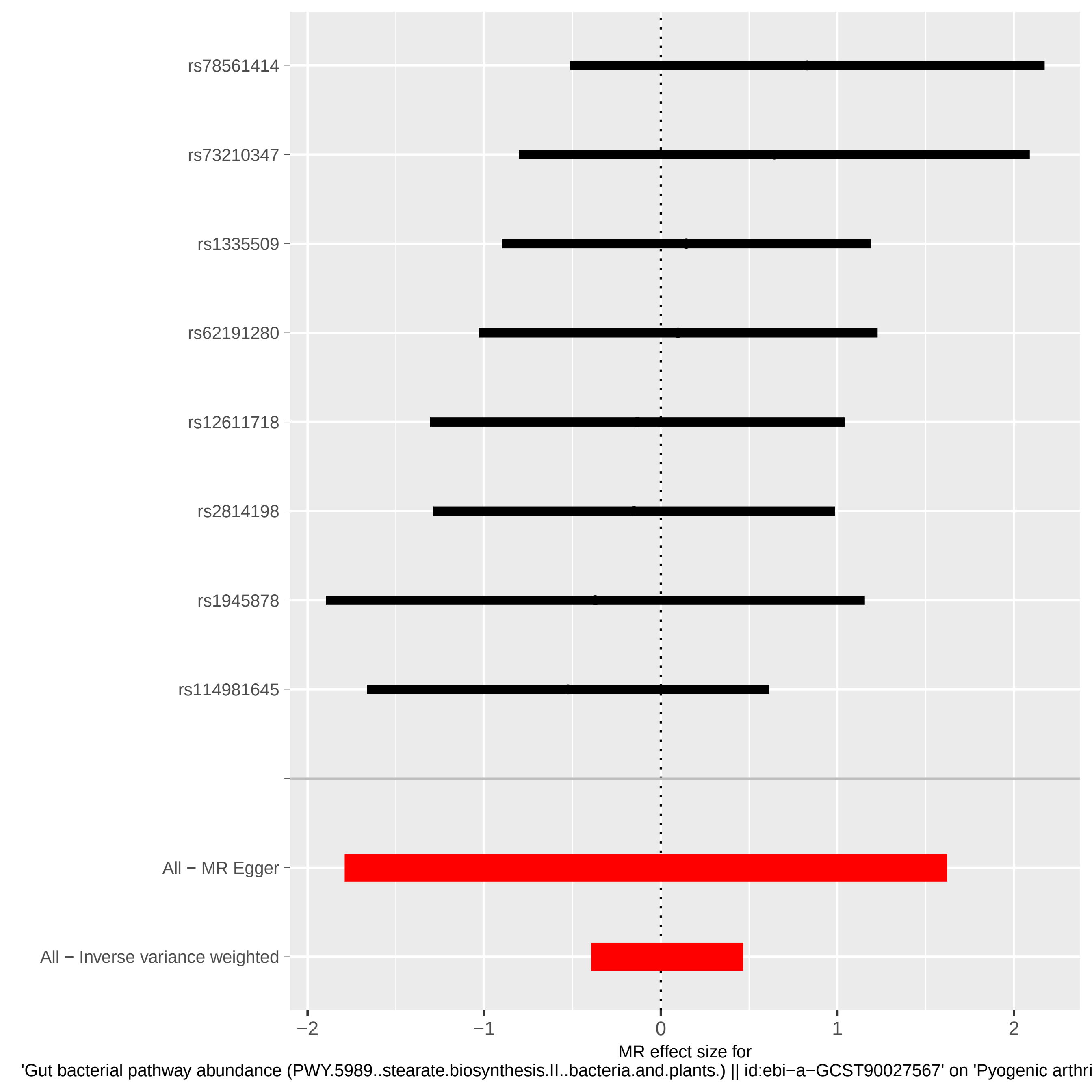

rs78561414
rs73210347
rs1335509
rs62191280
rs12611718
rs2814198
rs1945878
rs114981645
All − MR Egger
All − Inverse variance weighted
−2
−1
0
1
2
MR effect size for
'Gut bacterial pathway abundance (PWY.5989..stearate.biosynthesis.II..bacteria.and.plants.) || id:ebi−a−GCST90027567' on 'Pyogenic arthri
